# Legitimacy, Shared Understanding and Exchange of Resources: Co-managing Lakes Along an Urban–Rural Gradient in Greater Bengaluru Metropolitan Region, India

**DOI:** 10.1007/s00267-023-01795-z

**Published:** 2023-02-10

**Authors:** Arvind Lakshmisha, Andreas Thiel

**Affiliations:** 1 Section of International Agricultural Policy and Environmental Governance, Faculty of Organic Agricultural Sciences (FB11), University of Kassel, SteinStr. 19, 37213 Witzenhausen, Germany; 2 Centre for Climate Change and Sustainability, Azim Premji University, Burugunte Village, Survey No 66, Bikkanahalli Main Rd, Sarjapura, Bengaluru, Karnataka 562125 India

**Keywords:** India, Water co-management, Greater Bengaluru Metropolitan Region, Legitimacy, Shared understanding, Exchange of resources

## Abstract

Co-management is increasingly seen as a way forward in natural resource management and collective goods provisioning, especially in the management of urban commons. Co-management entails sharing of power between actors, including elements such as exchange of information and resources as well as changes in regulations favouring the development of common goals among actors. In this paper, we try to understand if and how preconditions of legitimacy, shared understanding and exchange of resources combine to facilitate the co-management of lakes in Greater Bengaluru Metropolitan Region (GBMR), India. To understand these issues, we undertook an exploratory, qualitative analysis of the governance of three lakes located within a single watershed placed along an urban-rural gradient. We provide an exploratory assessment of co-management across the cases situated in diverse contexts, highlighting the importance of heterogeneity of socio-economic settings for co-management of lakes. Community involvement in co-management varies with heterogeneity, correspondingly increasing transaction costs. In urbanising contexts, state actors have started to recognise the political efficacy of non-state actors mobilising knowledge and financial resources for lake management. Involvement of the state custodian and third-sector organisations (NGOs) was found to be crucial in developing and facilitating shared understanding. Deliberation between mutually dependent state and non-state actors was key to overcoming scepticism in order to realign actor perspectives. We highlight that increased acceptance of community participation based on the development of a collective identity and understanding of mutual dependence observed in our urban and rural cases reduced transaction costs and thus enabled co-management.

## Introduction

Co-management or collaborative governance systems are increasingly seen as a response to circumstances facing drawbacks of hierarchical state-led governance (Ansell and Gash 2007; Sandström et al. 2014), especially in the last decades due to declining budgets (Clark et al. 2013; Foster 2013; Sundeen 1985) and increasing awareness of the limitations of privatisation (Clark et al. 2013). Co-management is considered an alternative more frequently in urban regions due to diminishing enforcement and increasing non-compliance with regulatory standards when it comes to resource governance (Foster 2013). There are numerous definitions of co-management; all of them refer to co-management as a range of arrangements, with different degrees of power-sharing for joint decision-making by state and users (non-state) about a resource or an area (Berkes et al. 1991; Borrini-Feyerabend et al. 2004; Carlsson and Berkes 2005; Singleton 1998). The basic idea behind co-management is the need for an element of interaction between state and non-state actors through formal regulations and/or informal deliberations (Mees et al. 2018) that ensures actors’ right to decision-making regarding management of the resource (Carlsson and Berkes 2005). In this paper, we define co-management as *a partnership between state and non-state actors requiring direct and active contribution by all actors to ensure effective resource management*. From the perspective of public management, emphasis is placed on the partnership between stakeholders to achieve societal goals (Osborne and Strokosch 2013) attaining better quality, increased service satisfaction and public trust (Fledderus and Honingh 2016).

Co-management has developed as partnership arrangements, where non-state actors (communities and third-sector organisations) based on their capacities and interests complement the ability of the government in providing legislation, monitoring and enforcement (Pomeroy and Berkes 1997). This entails that not everyone is willing to collaborate; this depends on their motivation, trust and acceptance (Fledderus and Honingh 2016). Scholars have shown that actors actively participate when they understand why their engagement matters (Mees et al. 2017; Porumbescu et al. 2020). This understanding depends on individual capabilities and resources (human and financial), which has been shown to have a positive correlation towards the formation and support of co-management (Cheng 2019; Paarlberg and Gen 2009). Further, as pointed out by Ostrom (1990), the returns obtained when actors collaborate and coordinate their strategies to manage a resource are much higher than when they stay unorganised, which could then lead to the destruction of the resource. Scholars are increasingly focusing their research on understanding the determinants of co-management, the bulk of these studies focus on single case studies (Ansell and Gash 2007), with comparative case studies beginning to be undertaken recently (e.g. Sandström et al. 2014).

This paper attempts to understand how participation (legitimacy), shared understanding and exchange of resources among actors influence co-management using an exploratory analysis of three interconnected lakes along a rural-urban gradient and their comparison on an analytical level. We focus on lakes within the Greater Bengaluru Metropolitan Region (GBMR), as lake management has undergone significant changes since lake “ownership” was taken over by the state through passing of Karnataka Land Revenues Act in 1964. Over the years, due to limited budgets and rapid urbanisation, lake management was neglected, leading to leasing of lakes to private actors. This was highly criticised by concerned citizens and NGOs, taking a judicial recourse to protect and conserve lakes. These activities started off by informal groups of residents, they have developed into a network of groups that support through sharing experiences, advice and contacts (Enqvist et al. 2016). These groups advocate for greater participatory arrangements leading to some of them signing a memorandum of understanding with the city administration to share responsibilities of lake maintenance and monitoring (Luna 2014). This, coupled with a push for decentralisation by the Indian government, has led the state to accept participation of non-state actors in lake management in some cases, which forms the basic premise of this paper. Against this background our research question concerns what role the three variables of legitimacy, shared understanding and exchange of resources play in determining co-management. For the three categories of variables that we consider we find that each of them is necessary but not sufficient for co-management to emerge. Further, we find that salience of particular demands on the lake is crucial in motivating direct and active participation while necessary efforts (transaction costs) of organising for co-management are crucially determined by the contextual aspect of socio-economic heterogeneity of the community of users at stake.

## Conceptual Framework

**Fig. 1:**
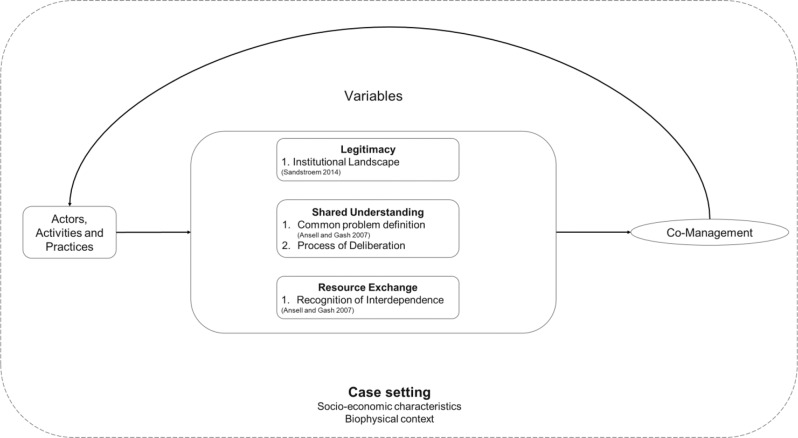
Framework used to understand the process of co-management along a rural-urban gradient (modified and adapted from Plummer et al. 2017)

In this paper, to explain our inquiries on how legitimacy, shared understanding and exchange of resources among actors influence co-management, we adapt and modify the framework for diagnosing adaptive co-management by Plummer et al. (2017) and the model of collaborative governance by Ansell and Gash (2007). The framework illustrated in Fig. 1 aids us in our enquiry of cross-case empirical questions of how the variables of legitimacy, shared understanding and exchange of resources (independent variables) facilitate co-management of lakes along a rural-urban spatial gradient. Socio-economic characteristics and biophysical context that shape activities and practices of actors involved are included as contextual conditions in the “case setting”.

We focus on both state and non-state (e.g. third-sector organisations such as community organisations, Non-governmental organisations) actors, in line with our definition of co-management, which highlights an engagement between state and non-state actors leading to collective action with direct and active contributions by all involved actors. Drawing on an extensive literature review on the determinants of co-management, we consider three main variables, namely, Legitimacy (Birnbaum et al. 2015; Sandström et al. 2014), Shared understanding (Ansell and Gash 2007) and Exchange of resources (Carlsson and Berkes 2005; Stoker 1995), to explain the presence or absence of co-management. In the process we acknowledge that, naturally, co-management can take shape in different forms. We are aware that these three variables can be considered interdependent. For example, legitimacy can be seen as an outcome of common understanding (Sandström et al. 2014). However, in order to ensure that we measure different things that do not necessarily follow from each other we operationalised the variables in a way that minimises overlaps and redundancies. Thus, we argue that independent observation of any of these variables in the way we operationalised them is well possible and that none of them is a sufficient condition neither for any other variable determinant of co-management that we investigate here nor for co-management itself.

## Legitimacy

Legitimacy defined as acceptance and justification of participation as indicated by Sandström et al. (2014) and Bernstein (2005), is considered an essential precondition central to collaboration (Jentoft 2000; Sandström et al. 2014) as it validates the representation and participation of societal actors (Hermans et al. 2021). As co-management is seen as a partnership, where state and non-state actors complement their ability based on individual capacities (Pomeroy and Berkes 1997), legitimacy plays a crucial role in enabling this partnership. This is especially due to increased push for decentralisation by policy makers and the communities themselves, especially regarding resource management (Cheng 2019; Foster 2013; Pomeroy and Berkes 1997). Among others, such a push for greater involvement of non-state actors requires establishing proper legal rights for them (Williams et al. 2016; Foster 2013; Pomeroy and Berkes 1997).

In this paper, following a normative view of legitimacy, we focus on the structures that lead to input legitimacy, which rely on participation (Johansson 2012). Input legitimacy focuses mainly on assessing if the extent to which the actors affected by decisions were involved in the process of decision-making. Our indicator is *Institutional Landscape*, which we measure as “existing institutions, organisations and collaboration structures” that cater to participation of state and non-state actors in the process of co-management (Sandström et al. 2014).

## Shared Understanding

Shared understanding is a crucial factor for stakeholders of co-management to identify a common purpose to work towards. As pointed out by Ansell and Gash (2007), shared understanding among stakeholders of what they can collectively achieve together is indispensable within a collaborative process. Shared understanding leads to what Mosimane et al. (2012) call collective identity. Collective identity among the members of a co-management group increases cost-effectiveness of co-management and cooperation by reducing transaction costs. As pointed out by Pahl-Wostl and Hare (2004), shared understanding implies an agreement on a definition of the problem, or might indicate the consensus on the necessary knowledge required to tackle a problem. Porumbescu et al. (2020), Mees et al. (2018) and Mees et al. (2017) indicate that actors are inclined to collaborate when they understand why and how their involvement helps achieve the outcomes. Scholars such as Pomeroy and Berkes (1997), highlight that shared understanding requires overcoming scepticism mainly among government officials “on the lack of appropriate knowledge and know-how on part of the users” reducing uncertainty and enhancing the credibility of non-state actors.

In this paper, we try to assess shared understanding among actors involved based on the indicators of *common problem definition* (Ansell and Gash 2007) and *process of deliberation*. We measure common problem definition based on the presence or absence of re-alignment (Sandström et al. 2014) of actors goals in the process of developing shared understanding. We measure the process of deliberation through presence or absence of ‘mutual communication that involves weighing and reflecting on preferences, values and interests regarding matters of common concern’ (Mansbridge 2015). It may or may not build on the institutional landscape but concerns the process of communication.

## Exchange of Resources

Resources play a critical role in co-management (Bovaird and Loeffler 2012) and community resources (human and financial capital) play a crucial role in increasing the likelihood of formation and support to their participation in co-management (Cheng 2019; Paarlberg and Gen 2009). Co-management relies on the idea that citizens represent a ‘huge untapped resource’ which can trigger innovation and assist in formation and support of collaborative relationships (Boyle and Harris 2009; Nabatchi et al. 2017; Paarlberg and Gen 2009). The importance of resources for co-management is founded within two theories namely, theory of power relations and theory of resource dependency (Carlsson and Berkes 2005). The power differentials due to asymmetries in resource allocation between actors is what leads to exchange and dependence (Johnson 1995). As emphasised by regime theorists, access to resources is what makes certain actors attractive for collaboration (Stoker 1995). Collaborative relationships are established to overcome lack of resources by an actor with those who have access to resources, and are successful when there is an understanding that the gains achieved by pooling individual resources are beneficial to all actors involved.

In order to measure exchange of resources, we defined the variable *recognition of dependence* which we measure through presence or absence of different kinds of Salience and Efficacy. Both these measures influence actors’ motivation to engage in co-management. Salience refers to “actors” perceiving a topic as important enough to consider active engagement and weighing their investment’ (van Eijk and Steen 2016). We focus on both personal salience, ‘individuals perception of how the service affects him/herself’ (Pestoff 2012 as quoted by; van Eijk and Steen 2016) and social salience, ‘perceived importance of the issue to one’s neighbourhood, community or society at large’ (van Eijk and Steen 2016). Efficacy refers to the perception of actors to make a difference. We use both personal (where actors believe that they themselves can make a difference) and political (where actors believe that people can make a difference) efficacy (Bovaird et al. 2016). Further, we consider aspects such as how actors managed a shortage of resources (such as funding, expertise and knowledge), through pooling of resources (Imperial 2005) among actors involved in the process to understand the influence of resource exchange on co-management.

## Material and Methods

### Case Selection and Description

Bengaluru is one of the five megacities^1^ in India, with an estimated population of over 13 million in 2022 (World Population Review 2022). There has been a massive increase in urban population from 44% in 1901 to 90.9% in 2011 (Puttalingaiah et al. 2019), resulting in rapid urbanisation of not just the city but the region as well, creating an urban agglomeration. The city covers a spatial area of 741 sq. km and the metropolitan region covers 8005 sq. km. The metropolitan region termed GBMR, spans over three administrative areas (Bengaluru Urban, Bengaluru Rural and Ramanagara districts). The drastic transformation of Bengaluru from agrarian context to an urban agglomeration during the last four decades was augmented by economic reforms and growing employment opportunities since the liberalisation of India’s economy in 1991. This urban transformation and economic development have had serious environmental impacts (Sudhira and Nagendra 2013). An analysis of the urban dynamics between 1973–2017 by Ramachandra et al. (2019) highlights 88% decline in vegetation and 79% decline in water bodies with increasing urban areas. The loss of water bodies and lakes is of particular concern for the region as there are no major rivers around the metropolis (Enqvist 2017). Lakes are man-made, by building of bunds and dams across small seasonal streams, along the undulating terrain of Bengaluru, which safeguarded the local communities to continue agriculture and rear cattle throughout the year (Nagendra 2016), in addition to regulating the micro-climate of the city. In order to minimise degradation of lakes across the region various citizen groups have started to collaborate with state authorities, with varying levels of participation. Thus, GBMR provides us with a living laboratory to undertake research in our effort to understand co-management in an urbanising local environment.

We use multiple contrasting case studies (Yin 2018), allowing us to compare the process of co-management of three lakes in GBMR. On an analytical level, this implies that we can identify underlying common abstract variables driving co-management rather than drawing conclusions from direct comparison of observations. Direct comparisons are difficult because of the great difference in the context of the lakes. The selected lakes differ in their geographical location, population density, socio-economic and biophysical characteristics highlighted in Table 1. The variations of socio-economic variables and population densities are aligned with our corresponding expectations along an urban-rural gradient with population densities being greatest in urban areas, less dense in the peri-urban area and least in rural areas while socio-economic heterogeneity, for example, is greater in the urban and peri-urban area in comparison to the rural area. The lakes are located within a single (Vrishabavathi) watershed, which is a major outlet for both domestic and industrial wastewater, converting a once seasonal stream into perennial source, ensuring continued agriculture-based livelihood (Jamwal et al. 2014; Lele et al. 2013).

**Table 1:** Socio-economic characteristics of communities adjacent to lakes and biophysical characteristics of the lake across the three cases

	Socio-Economic Characteristics			Biophysical Characteristics			
	Population Density (number of people living per square kilometre)	Literacy Rate (%)	Predominant livelihood	Dependence on the lake for livelihood	Ecosystem services derived from the lake	Water Quality	Water Quantity
Urban Lake	6218	76.4	Diverse urban livelihoods	Low	Cultural and Recreational Services	Wastewater is diverted from the lake	Regulated
Peri-urban Lake	Average across the four villages in 516.43 (highest is 791 and lowest is 297)	Average for four villages 66.61 (High 72% and low 60%)	65% in three villages and 12% in the fourth village engaged in agriculture and allied activities	Mixed	Production based services	Wastewater from upstream urban and industrial areas	Unregulated leading to abundance
Rural Lake	391.28	63.6	69.6% depend on agriculture and allied activities	High	Production based services	Wastewater from upstream urban and industrial areas	Regulated

Below, we provide a brief description of the three selected lakes, highlighting their socio-economic and biophysical characteristics, thus describing the case setting, which is summarised in Table 1.

#### Urban Lake

located in one of the most densely populated areas of the city, the lake was created in 1869 for irrigation (EMPRI 2018). The socio-economic characteristics changed as the area around the lake grew tremendously since 2001 with a decadal population growth of 161.9% and a household growth of 176.3% (2001–2011) (Census 2011). The residents comprise of heterogeneous community, consisting of people from diverse economic, social and educational background (refer Table 1) speaking diverse languages and working in private or public sectors. The new residents were unaware of the lake nearby or its benefits to the local ecology; hence, they never connected to the lake as compared to older residents. Biophysical context highlights that the lake is no longer a recipient of wastewater, since its restoration by the city administration in 2009–10, as water inflow was modified by diverting wastewater away from the lake. The citizens derive recreational and cultural services, as they are not dependent on the lake for their livelihood. The city administration in 2010 signed a Memorandum of Understanding with a third-sector organisation to ensure day-to-day management of the lake.

#### Peri-Urban Lake

Located downstream from the city, the peri-urban lake was expanded in 1946 increasing its capacity to irrigate fields belonging to four villages. Biophysical context of the lake transformed with inflow of upstream wastewater (domestic and industrial) since late 1990s, converting it into a perennial lake. The role of the community has dwindled greatly since the official takeover of lakes by governments in 1964. It was abandoned *with the conversion of the lake into a perennial source*” of water. Socio-economic characteristics of the actors vary across the villages (Table 1) in the area, with the lake being an important source of livelihood in three of the surrounding villages as 65% of the population are dependent on agriculture and agricultural labour for their livelihoods (Census 2011 and corroborated during interviews). The fourth village is under the jurisdiction of the nearest town and has an industrial estate located along the banks of the lake. This has resulted in decreased dependence on agriculture with only 12% being engaged in agriculture (Census 2011) due to possibilities of new opportunities as indicated by community members during group discussion.

The state stopped collecting irrigation water cess^2^ in 2000 (Bangalore Environment Trust 2021). The reasons were increased levels of pollution, prompting the state to give up monitoring and enforcement of regulations for lake management. In order to manage the water quantity of the lake, the state custodian expanded irrigation channels creating new users who consequently started to have a say in lake management.

#### Rural Lake

The rural lake is a recipient of the outflow of wastewater from peri-urban lake. This has transformed the biophysical context by increasing water quantity thus, converting a seasonal lake into a perennial source. The availability of (polluted)water has maintained the community intact, and provided stability to socio-economic characteristics of the rural communities, with a majority 69.6% of the population dependent on agriculture and associated activities (agricultural labour and dairy industry) as their main livelihood (Census 2011). The lake water is used to irrigate four crops a year, (as indicated during interviews and corroborated during field visits). There is a trade-off between economic benefits over health issues by the community, leading to minor differences among members, resulting in construction of another lake in 2014 along the wetlands collecting clean water in the village (this lake is not the focus of this paper).

### Data Collection

Qualitative data was collected through key informant interviews and focus group discussions, conducted in 2018–19. Purposive sampling was undertaken to identify respondents, classified into state and non-state actors. We identified non-state actors by visiting the lake and talking to residents identifying key members of the community or third-sector organisations involved in lake management. Documents and interviews led to identification of state actors. Following our definition of co-management, we focused on actors actively involved in day-to-day management of the lake, which led us to identify designated state custodians who had a direct role in lake management. Thus, we narrowed down from a large mosaic of state agencies responsible to two main state custodians and the views of officials was considered to be representative of the agency.

Key informant interviews were held with state officials across the three cases (*N* = 5 custodians; *N* = 5 officials of local administration in peri-urban and rural), representatives of the citizen groups in urban case (*N* = 5), researchers and academics (*N* = 4), representatives of NGOs (*N* = 7). Focus group discussions were undertaken in rural (*N* = 2) and peri-urban (*N* = 5) communities. The number of focus group discussions in the peri-urban case is greater due to the presence of four villages along the lake. Three of the four villages are dependent on the lake for their livelihood, whereas the fourth village comes under the administrative jurisdiction of the nearest town and is home to an industrial estate, with limited agriculture. During interviews and discussions, respondents were asked about their role in lake management, presence of platforms for participation, practices of stakeholder participation, acceptance, and openness towards inclusion of local knowledge, reasons for collaborations. We also reviewed data from secondary sources, such as formal laws, policies, rules, and regulations in addition to research and academic contributions. The interviews and discussions lasted between 45 min to 2 h, were transcribed and coded using Nvivo.

## Results

In this section, we explain the presence or absence of co-management based on the above framework. Accounts for each lake first describe actors, their activities and practices, the observation of variables of legitimacy, shared understanding and exchange of resources before we assess presence or absence of co-management and describe its particular form.

### Urban Lake

#### Actors, actions and practices

There are three main actors, directly and actively involved in managing the lake, namely: City administration (State custodian, henceforth BBMP), United Way Bengaluru an NGO (henceforth UwB) and local community association (henceforth, LCA). We first describe the actions and practices of the state actor followed by the non-state actors.

BBMP as the state custodian is responsible for decisions regarding day-to-day management, including removing encroachments, maintaining bunds, embankments, and the area around the lake. In 2010, it invited UwB to sign a MoU to secure finance from private actors and organise the heterogeneous local community by creating awareness. The MoU defines BBMPs role as provider of infrastructure (embankments, bunds…) needs, in addition to ensuring that no sewage and chemical pollutants enter the lake.

UwB plays a crucial role in securing financial support for lake management. They are known across the city for securing corporate funding, under corporate social responsibility schemes specifically for social issues. As per the MoU, they are also responsible to create public support and generate public participation in activities concerning the lake. They organised a heterogeneous community into a local association, who were made a signatory to MoU in 2017. UwB organises numerous activities, by working with local elected representatives and community leaders involving both corporate volunteers and local residents. These activities (information-sharing events, tree plantations, educational walks for local schoolchildren and so on) have led to exchange of perspectives and alignment of values, among actors.

The LCA is responsible for general housekeeping activities of the lake. According to the MoU, they are responsible for providing security, maintaining the area free from garbage and monitor encroachment. According to community members, “LCA has become the face for the community and help in information exchange between the residents and other actors involved”.

#### Assessment of pre-conditions of co-management

##### Legitimacy

The institutional landscape for participation in urban areas is enshrined in the 74th Constitutional Amendment act, which mandates devolution of power to city governments (Urban local bodies) establishing and empowering ward^3^ committees (Interview TS3, 2018). Any citizen may approach the committee for addressing issues related to public and ward development and the committee is obliged to meet once a month (Karnataka Gazette 2016). According to community members “Though there is the provision for ward committee, its establishment has been slow, and committees are not even formed.” The same is the case in our urban lake as indicated by a member of LCA “we are unaware of the ward committee…”

In our case, we see that formal participation is enshrined in the tri-partite agreement signed between the state and non-state actors. It obliges the signatories to ‘meet regularly’ and discuss implementation of individual roles and responsibilities (BBMP 2017). Formal rules of participation were followed as informal rules of participation were not established among actors.

##### Shared understanding

There is shared understanding among actors developed through the intervention of UwB, who was responsible for generating public support as indicated in the MoU. In this regard, UwB as an outsider had to gather the support of heterogeneous residents by realigning community perceptions of the lake and its management. According to a member of an NGO “The new residents had come to see the lake as an eyesore of the neighbourhood” and had no understanding of the important of the lake to the local ecosystem; the older residents distrusted state intervention. As indicated by members of the NGO “UwB struggled for nearly 6 months to get the community to participate in activities related to the lake.” A change and alignment of attitudes among residents were achieved through campaigns, activities to clean the lake and working closely with the local elected representative, gaining support of local leaders to gain the trust of the community. These got residents talking about the lake (common problem definition through realignment of community goals) and they “started to enlist their support in collaborating with UwB…” as indicated by member of NGO. According to a city Official, “these activities led to consideration of UwB as a trustworthy partner” and seriously considered suggestions put forward by UwB. Further, as indicated by members of UwB, these activities helped realigning community perceptions of state apathy in lake management, motivating and community members to participate in lake management. UwB organised and established LCA to monitor day-to-day activities such as cleaning, maintenance. The process of deliberation between actors is outlined in the MoU. The actors are obliged to meet once a month to discuss issues, according to members of LCA “we meet once a month to discuss issues and reflect on concerns raised before deciding”.

##### Resource Exchange

There is a recognition of dependence among actors involved. Researchers and community highlighted that, the state custodian recognises the importance of third-sector organisations to complement state financial support through corporate social responsibility funds and organise the community, highlighting political efficacy. Beyond efficacy, recognition of dependence is illustrated by pooling of resources by non-state actors in the form of securing financial support from corporates and knowledge sharing. As indicated by members of UwB, “every lake is unique and local knowledge plays an important role, [thus] we work with communities to understand the local geography and ecology before planning actions”.

The community considers active participation by actors in lake management to lead to betterment of the neighbourhood (social salience). As indicated by community members “the lake has been transformed into a social space and this also has a positive influence on the real-estate value” UwB follows its organisational motto of working with communities by listening to their concerns and empowering them to act in order to overcome problems. Thus, viewing lake management as crucial aspect of society and believes that people can make a change.

##### Outcome

In the urban case, we can clearly observe co-management. UwB adopts an active role in organising a heterogeneous community on behalf of the state custodian in addition to securing finance from corporates. Even though UwB was invited by the state, its activities have overcome state scepticism previously held by the community. Actors have learned to consider each other as trustworthy partners. This has led to pooling of knowledge and finances and community understanding that lake is an essential part of the neighbourhood.

### Peri-Urban Lake

#### Actors, Actions and Practices

For the peri-urban lake, we identified, the Minor Irrigation Department (State custodian, henceforth MiD) and non-state actors (communities)—from villages around the lake (traditional users), among whom we distinguish from new user communities who use lake water through irrigation channels. We first describe the actions and practices of the state actor followed by the non-state actors.

MiD is responsible for “decisions regarding management, monitoring and enforcement”, as indicated by an official. The main objective of MiD is to provide water for irrigation from the lake. Thus, they have extended irrigation channels to irrigate farmlands up to 12 km from the lake. The officials no longer monitor or enforce regulations due to high volumes of wastewater inflow.

The community lacks any form of authority and willingness to get involved in lake management due to two main reasons; first, economic benefits obtained from irrigating the land throughout the year have offset the ill effects of wastewater on not just their health, but cattle and soil. Second, the increase in the users, with expansion of irrigation channels. This was done without consultations with the traditional users, which has increased distrust towards the state among traditional users, causing a rift among user communities and inhibiting cooperation among them. Further, while traditional users are willing to contribute to lake management, the new users who fear losing rights to water once the current management regime is modified.

#### Assessment of pre-conditions of co-management

##### Legitimacy

As regards institutional landscape, the structures for participation in rural India are enshrined in the Indian constitution through the 73rd Amendment in 1992, realised through Panchayat Raj Institutions. These consist of institutional structures that devolve powers and responsibilities to village organisations, namely the gram panchayat (village council) which represents the community through direct elections and the Gram Sabha (village meetings) which addresses planning for economic development and social justice (Das 2022). Gram Sabha is the prominent structure that provides for participation of all adults registered in the electoral role of the village (Das 2022). The village council is obliged to hold at least two general meetings per year, to discuss development plans, budgets allocated under various policies and so on.

In our case, the villages around the lake came under diverse jurisdictions (two village councils and one town council), which organise meetings within their respective boundaries. This institutional landscape of participatory structures has caused confusion as to who is actually responsible for the lake, increasing animosity between communities. Though the Karnataka Panchayat Raj Act 1993 provides a mechanism for setting up a joint committee between panchayats to solve issues of common purpose based on joint interest, there has been no joint interest shown by the panchayats or the communities. The members of village councils indicated, “If the lake was within one village and we had control over the inflow of water, we could do something about it. But the water comes from somewhere and is utilised by villages up to 12 km… it is difficult to manage it locally.” Thus, ultimately the indicator of institutional landscape for co-management is not observed in the case.

##### Shared Understanding

There is no shared understanding between and within state and non-state actors, explained by the difference in perceptions of the community as to how the state custodian is managing the lake. Traditional users (community) perceive that officials are not interested managing the lake reflected by their statement “we have been asking for the betterment of the lake, but the officials are not showing any interest”. In contrast, new users are content with lake management, respectively the absence of its management. This has created distrust among users and between traditional users and state actors, further amplified by the lack of knowing “who” to approach. This has been, summarised by community member “… they say an engineer is responsible for the lake, but I have not seen him till date…” Further, diverging views on water quantity also led to lack of common understanding between traditional and new users and the state. The state custodian follows its organisational vision of providing irrigation facilities to maximise utilisation of wastewater, whereas traditional users request for a “reduction in the water quantity of the lake.” In contrast, new users are sceptical about any changes, and try to ensure continuation of existing practices. We see absence of common problem definition in this case.

Platforms for deliberation are seen as top-down information sharing and these platforms do not fulfil the characteristic of aggregation of stakeholder preferences. There is no platform for interaction between non-state actors leading to decreased trust and social capital, as indicated by community members “Byr is now a different panchayat, they will get some things approved and they will use the money themselves.” Traditional users indicated that the addition of new users without consultation has “increased diversity and decreased trust between state and non-state actors.” Further, water quantity is a cause of concern as indicated by both state and non-state actors. According to members of village councils and community, “…existing local institutions, organisations and structures are unable to handle the situation and it requires interventions from higher authorities.” Thus, we consider processes of deliberation as absent in this case.

##### Resource Exchange

There is no exchange of resources between actors explained by lack of salience and efficacy. Members of the community perceive the economic benefits of cultivating four crops a year to be higher than actively engage in lake management. Traditional users indicated that, they would not do anything to harm the new users, as “they are farmers too, they are dependent on the lake just like we are, and we would not want to steal their livelihood.” Thus, indicating importance of the lake to be much larger than the village boundaries, prompting their non-involvement in lake management (social salience). Community within one village under the jurisdiction of the town administration did not want to be associated with the lake, as they indicated, “…we do not have any use of the lake as we are not dependent on it.” Further, expansion of irrigation channels has led traditional users to perceive that they themselves cannot make a difference (political efficacy) as indicated during discussions “lake water is used by villages for at least 12 km… so it is now not easy for one person or village to do anything.”

##### Outcome

There is no co-management of the lake mainly due to diverging perceptions among actors as well as increasing dependents due to water availability. Expansion of irrigation channels by state to manage water quantity has led to an implicit recognition of dependence among actors from the perspective that lake management needs involvement of higher authorities.

#### Rural Lake

##### Actors, Actions and Practices

We identify two main actor groups, actively and directly involved in lake management namely: MiD (state custodian) and community (non-state actor). The MiD is the designated custodian and performs the activities as in the peri-urban case. Further, MiD is expanding channels within the village administrative boundaries to provide irrigation to farms based on consultation with community member, as channels need to pass through their fields.

Community has had to reclaim its role in lake management since it became a perennial source. To overcome the ill effects (reduction in crop productivity, human health) of using wastewater, the community gathered information to identify alternative practices and ensure crop productivity, leading to changes in cropping patterns (cash crops and fodder for cattle). This need for information fuelled the community to identify a “field officer” in 2014, and to liaise and collect information regarding state policies, regulations and rules (including lake) that are beneficial and share it with them.

#### Assessment of pre-conditions of co-management

##### Legitimacy

The Indian constitution provides the structures establishing participation in the form of local self-government of villages. Similar to the villages in the peri-urban areas, these institutions established under the Karnataka Panchayat Raj Act 1993, provide the legal democratic structure and are tasked with administrative, socio-economic functions, including construction and maintenance of ponds. The Gram Sabha is crucial in providing a platform for participation for all adults residing within the boundaries of villages (Das 2022), establishing a formal platform for participation, where officials are obliged to present their plans for development and have discussions with those directly affected.

In our case, as highlighted during interviews and discussions with members of the community, ‘the Gram Sabha meets once in 6 months’ as stipulated by law under the Karnataka Panchayat Raj Act 1993. The actors discuss issues related to overall village development and the topic of lake management is key. As indicated by community members, “the community gains most of its information from the panchayat meetings, as all the department officials are present and inform us of various schemes by the government.” Correspondingly, we consider the precondition of institutional landscape as being met.

##### Shared understanding

In the rural case, the state custodian, following its departmental vision of adequate use of water bodies (Minor Irrigation Department 2022) focussed on providing and expanding irrigation not just to new areas but also to develop existing agricultural lands. State actors viewed communities as ignorant of technical issues and did not consider the community, as there was no formal need to involve non-state actors in lake management. The community did not participate in issues of management as they considered the economic benefits of cultivating four crops. Community members indicated during discussions that “wastewater is of great help to us the farmers, we can grow crops round the year, and we do not have to spend money on fertilisers and pesticides.” This perception of the community started to change with increasing awareness of ill effects of using wastewater highlighted during discussions with “not only people are falling ill, but even cattle are also dying…” Thus, the community felt the need to [re]align not just their views of the lake as source of economic well-being but it cohered on a more critical stance towards state officials, who were involved in provision of irrigation channels and did not consider the views of the community or the quality of the water. This [re]alignment of community goals by members can be explained by two reasons: first, the lake is main source of livelihood and has negative impacts on health, as highlighted by the community ‘lake is the most important source of our livelihood as a majority depend on agriculture.’ Second, inspiration drawn from media and news stories of community management of lakes in the (upstream) city, which led to the decision of the community to get itself involved.

In 2014, the urge to participate made the community identify a community member as “field officer” who would liaise with state departments and collect information. As indicated by members of the community, this allowed for better-informed “discussions at village meetings and presenting their case to state actors.” These discussions made state officials take community views seriously (realignment of state goals) which led to increasing community role in regulation of water quantity of the lake. Both state and community actors highlighted that, informed mutual discussions based on information collected and local knowledge has resulted in building of social capital overcoming state scepticisms. Correspondingly, over time, a process of deliberation became observable. As indicated by all actors, state actors have started to view the community as knowledgeable and consider their views. Thus, the community initiative to better liaise with state actors led to realignment of goals regarding the lake. Thus, the variable of common problem definition was achieved.

##### Resource Exchange

Community recognises its dependence on the state for funds and technical knowledge, as highlighted by members “we lack the technical skills and appropriate knowledge and finances for undertaking large scale efforts of lake management (building bunds, pitching embankments…) these can be complimented with local ecological knowledge within the community.” The community contributes own resources when state funds are insufficient to meet the goals of lake management (pooling of resources). Community members highlighted during discussions “we take government money and when that is insufficient, we collect from the village… the price per household is decided at the village meeting… if households are unable to provide money, they can volunteer to provide manual labour.”

This recognition is based on community understanding that their active engagement is crucial for their household income, as the lake is their main source of livelihood. The community views their engagement in lake management to be beneficial to them as well as the village, indicating personal and social salience of lake management. This was indicated during discussions with community members “water should reach all fields in the village… many people offered parts of their lands to build channels… we are all farmers we understand the plight of others who do not have direct access to water.” The community has come to see that government alone is unable to do things and they play an important role in lake management. Community members indicated that they had seen the “lake deteriorate over the years due to state apathy and inflow of wastewater” and “we cannot blame only the government, even in our own village we are losing community attitude and behaviour… of working towards the betterment of the village.” Thus, featuring both personal and political efficacy.

##### Outcome

In this case, we observe co-management initiated by the community. The community changed from not participating in lake management to co-management because of its increasing awareness of the ill effects of wastewater and understanding the importance of their participation. They gathered information to interact with officials in village meetings, (common problem definition) and engaged in a process of deliberation leading to co-management.

## Discussion

As expected, we found difference in constellations of co-management of lakes in GBMR and different ways in which legitimacy, shared understanding and exchange of resources were brought about. Table 2 summarises our findings. Specifically, we found a mode of co-management led by a third-party organisation on behalf of the state in the urban case and a mode of co-management led by the community in the rural case. In the urban case, the third sector organisation significantly lowers the transactions costs of cooperation of a relatively heterogeneous community. Despite limited benefits perceived by some members of the community, that way co-management is induced through development of a shared understanding. In the process, particularly state officials recognise the political efficacy of the community and the value of mobilising its knowledge of the lake. Co-management therefore emerges as a result of significant initial investments into lowering its transaction costs. This matching of the difficulties of co-management in heterogeneous social-economic contexts is made possible by exogenously provided CSR funds that are mobilised by the third sector organisation. Together with the openness of the state in that regard, we consider this the crucial factor making co-management come about. It leads to the creation of a shared understanding and the recognition of the need for exchange of resources.

**Table 2:** Table summarising the influence of legitimacy, shared understanding and exchange of resources on co-management across the three cases

Variables	Indicators	Measures	Urban Lake	Peri-urban Lake	Rural Lake
Legitimacy	Institutional Landscape	Structures that cater for the participation of state and non-state actors in the process of co-management	Formal participations structures provided by the 74^th^ Constitutional Amendment and MoU between actor groups are practiced as a result of UwB involvement	Formal structures provided by the 73rd Constitutional amendment are practiced within administrative boundaries	Formal participatory structures provided by the 73rd Constitutional amendment are practiced in context of Gram Sabha
Shared Understanding	Common Problem Definition	Realignment of actor goals	A common definition is created as a result of the engagement of a third-sector organisation promoting community participation of community and exchange with state agents	Heterogeneous values and perceptions regarding lake management with no common definition for lake management	Community led realignment as a result of better information with introduction of a liaison officer for state engagement
	Process of Deliberation	Mutual communication and reflecting of preferences and values	Detailed in the MoU signed between actor groups leading to overcoming state scepticism	No mutual communication between and within actor groups	Exchange leads to community coherence, informed engagement with state and overcoming state scepticism
Exchange of Resources	Recognition of dependence	Salience (Personal and Social salience)	Social salience recognised by the community as a result of UwB engagement	No perception of salience by actors	Personal and social salience due to dependence on the lake
		Efficacy (Personal and Political efficacy)	Recognition of political salience of community involvement recognised by officials as a result of UwB engagement	No perception of efficacy by actors	Personal efficacy as community feels it can make a difference
		Pooling of resources	Pooling of resources by securing financial support from corporates and knowledge sharing facilitated by UwB	No pooling of resources	Pooling of knowledge by community and state
Mode of Co-management			Co-management led by UwB on behalf of the state	No co-management	Co-management initiated by the community based on dependence on the lake

In contrast, in the rural community, co-management is triggered by salience to the community and the underlying realisation that community and the state interdepend in relation to lake management (exchange of resources). In a farming community water management is of core importance making it personally salient because it is decisive for individual livelihoods. Further, in the relatively homogenous socio-economic context the rural case investigated community benefits to add social salience. Thus, over time, water management for agriculture as well as water quality issues become important to an extent that the community invests in developing coherent and better-informed positions vis-à-vis the state. This in turn leads to better-aligned perspectives internally and better exchange with state authorities developing shared understanding (aligned incentives) through processes of deliberation. This makes state authorities come on board and better cooperate with the community and better align its preferences. The expected benefits of co-management trigger the community to invest in this case.

Finally, in the peri-urban case development of co-management is riddled with several obstacles. The socio-economic context is heterogeneous in several ways, making cooperation within the community difficult. In fact, for new users it becomes personally salient to not engage in co-management but to defend the status quo of extensive water provisioning. The extent to which co-management compromises livelihoods for traditional users could not be established but gaps in perspectives between new and traditional users seem to be unsurmountable. Further, stakeholders to co-management in the peri-urban case seem to be unclear as much as responsibilities of public actors are not clear. This leads to a lack of commitment among public actors to engage and it makes effective co-management even more difficult because of increasing transaction costs.

Altogether, these findings confirm that it is diverse context conditions that explain the pathways that lead to differences in co-management (Armitage et al. 2008; Husain and Bhattacharya 2004). Although more detailed reconstruction of cases leads to a kind of sequential argument about the relevance of the three variables of shared understanding, exchange of resources and legitimacy, we found that all three are vital for co-management to emerge. Further, although we measured different things for these variables, we found that they still largely condition each other. Thus, we conclude by proposing legitimacy, shared understanding and exchange of resources in the way we operationalized them are three necessary and together are also sufficient conditions for active and direct contributions by all actors to lake management.

Finally, the setting seems to play an outstanding role for the emergence of co-management or its failure more in general. Heterogeneity of the community affects preferences of its members and found to be to be of overriding relevance. It affects co-management in two ways. First, it affects social salience as actors will only engage into creating benefits for the community if they cherish it, we expect. Second, it affects transaction costs of coming to an agreement on the position and engagement of a community. Thus, costs of co-management significantly rise if the socio-economic context is heterogeneous, we expect based on the urban and peri-urban case. Ray and Bhattacharya (2011) also highlight that heterogeneity increases transactions costs by lowering costs of cooperation. If the community was left to its own devices and incentives to improve the situation were insufficient, co-management will not result. Finally, this intricate relationship between costs and benefits of co-management is context dependent also in relation to how contextual factors shape perceptions of livelihood threats that emerge from its absence. These seem to be side-lined in the peri-urban case. Correspondingly, lack of understanding of the local ecology and threats to livelihoods lead new users in the peri-urban case to discard co-management.

In what follows we want to further reflect on the relevance of the variables we investigate in relation to the literature and the particular Indian context.

Shared understanding among the actors as to why and how their participation matters is key for active contribution by actors across all three cases as indicated by Mees et al. (2018). The diverse perceptions of the lake have led to differing definitions of problems associated with the lake. As exemplified by our peri-urban case, each community is driven by lack of awareness and self-interest do not engage with each other (cf. Sharp 2012). In the peri-urban case this is amplified by a lack of deliberation process increasing scepticism between not just state and community but within the community as well (cf. Clark et al. 2013). In contrast, information gathering by community in the rural case created awareness and helped develop a common appreciation among all actors concerning the importance of the lake and decreasing scepticism as indicated by Thieken et al. (2007) which realigned community and state perceptions as also documented by, Sharp (2012) and Bohensky et al. (2010). In the urban case, increasing direct participation among actors overcame state and community scepticisms leading to shared understanding. This has led to the development of a collective identity as identified by Mosimane et al. (2012) and reduced the transactions costs for co-management.

The institutional landscape providing structures of legitimacy through community participation is enshrined in the Indian Constitution, in the form of local self-governance but the quality of its implementation varies across the cases. In accordance with Rajashejkar et al. (2018), we attribute lack of community participation in urban case to a lack of active ward committees. Further, we observed that initial lack of interest among communities in our urban and peri-urban cases is fuelled by evidence of deterioration of lakes, which has undermined communities’ willingness to participate and decreased trust in the state, as highlighted in the study by Fjeldstad (2004). Lack of information and knowledge about lakes and their role in the urban fabric among urban and peri-urban communities and the responsible agencies increased communities’ transaction costs of engagement, which could only be overcome in the urban case, given the availability of CSR funding.

The realisation of the need for exchange of resources between actors is crucial in co-management, as indicated by Stoker (1995). Actors across cases tend to collaborate with those who have access to resources, they do not possess. The urban and the rural cases highlight the mutual dependence between state and community in terms of knowledge and financial support. The state as the custodian of the lakes is a crucial actor. Thus, as indicated by several scholars such as Pomeroy and Berkes (1997), Sharp (2012) and Clark et al. (2013) overcoming state scepticism is key for co-management, which has been achieved in both rural and urban cases. In contrast, there is no recognition of dependence in our peri-urban case as actors distrust each other because of increasing heterogeneity and lack of personal efficacy.

## Conclusions

In this paper, we present an exploratory assessment of co-management in three cases situated along diverse contexts of urbanisation. We compared them on an analytical level with the aim to understand the relevance of three variables for emergence and functioning of co-management. Though the cases are not strictly comparable, based on qualitative evidence we found heterogeneity of the socio-economic setting and salience emerge as important aspects influencing co-management across cases. The role of the community in co-management is seen to vary with increasing heterogeneity of communities. Greater state involvement is required to facilitate co-management, in urban areas as communities become heterogeneous. We identify a homogenous rural community, who depended on the lake, directly engaging with state actors whereas the development of shared understanding had to be facilitated by an NGO in the heterogeneous urban community. Further, we find that actors engage with each other based on the importance they associate with the lake, which is captured by salience. Contextual factors which determine the possibilities of alternative livelihoods greatly matter here. This highlights the combined importance of socio-economic heterogeneity (high in urban case, low in rural case) and personal salience (low in urban case and high in rural case) for co-management. These findings are confirmed by the contrasting peri-urban case. Here salience (both personal and social) is relatively low and socio-economic heterogeneity high, which, in the absence of shared understanding leads to the absence of co- management.

As limitations of the study, we need to acknowledge that we look at a highly restricted set of explanatory variables in this paper. Each of the variables considered is multi-faceted and is dependent on numerous social, political and economic factors, which are beyond this paper. Further, we focus only on those actors who are active and directly involved in lake management overlooking others and providing just a snapshot of the actual realities on ground. Also, we did not address the environmental effects of co-management on the setting and the actors themselves.

Our results indicate that none of the three variables are individually a sufficient condition for facilitating co-management in the region but, all three are necessary together. The presence of structures for participation, though very important, do not ensure participation, actors need to realise the importance of their participation to ensure co-management. Further, we highlight that although the reasons for engagement differ across cases, a shared understanding along with a process of deliberation among actors is crucial for co-management to be present. An understanding that lakes have a societal impact in addition to personal benefits augments dependence among actors. Third-sector organisations are crucial in organising a heterogeneous community around a common problem definition and facilitate state engagement. This leads to institution building by developing both vertical and horizontal linkages between and within actor groups as is seen in the urban case. We conclude this study of three illustrative cases by indicating that there is a need to expand the study of the relevance of the three variables investigated in this study for example, addressing medium-level of n and using different methodologies such as qualitative comparative analysis to understand co-management.

## Footnotes

1 Any city with a population of more than 10 million is termed Megacity by UNDESA (2019)

2 Water cess collected as a means of betterment contribution from those who were benefiting from the irrigation work ensuring the government maintains channels and other infrastructure.

3 Ward committees are the lowest administrative unit of the city and are determined by population, geographical condition and economic status
